# Supplementary material for: A workflow for segmenting soil and plant X-ray computed tomography images with deep learning in Google’s Colaboratory
Source: Front Plant Sci. 2022 Sep 13;13:893140. doi: 10.3389/fpls.2022.893140 (PMC9514790; doi:10.3389/fpls.2022.893140)
Supplement: Supplementary file 1 [file Data_Sheet_3.pdf]

SI Table 2: Tabular representation of the sample types, scale, unique scan number, training/validation image number, and testing image number used to generate various models use in the paper.

Sample Type	Scale	Unique Scan #	Training/Validation Image #	Testing Image #
Walnut Leaf	1	1	5	5
	1	2	10	5
	1	3	15	5
	1	4	20	5
	1	5	25	5
Almond Bud	0.5	1	7	7
	0.85	1	7	7
Soil Aggregate	0.5	1	11	5
	0.85	1	11	5

SI Table 2: Tabular representation of a Pytorch implementation of a Fully Convolutional Network with a Res-net 101 backbone with an input image size of 1000x500 pixels with 6 material classes.

Layer (type:depth-idx)	Output Shape	Param #
FCN	[1, 6, 1000, 500]	--
├─IntermediateLayerGetter:	[1, 2048, 125, 63]	--
│ └─Conv2d:	[1, 64, 500, 250]	9,408
│ └─BatchNorm2d:	[1, 64, 500, 250]	128
│ └─ReLU:	[1, 64, 500, 250]	--
│ └─MaxPool2d:	[1, 64, 250, 125]	--
│ └─Sequential: 1	[1, 256, 250, 125]	--
│ │ └─Bottleneck: 0	[1, 256, 250, 125]	75,008
│ │ └─Bottleneck: 1	[1, 256, 250, 125]	70,400
│ │ └─Bottleneck: 2	[1, 256, 250, 125]	70,400
│ └─Sequential: 2	[1, 512, 125, 63]	--
│ │ └─Bottleneck: 0	[1, 512, 125, 63]	379,392
│ │ └─Bottleneck: 1	[1, 512, 125, 63]	280,064
│ │ └─Bottleneck: 2	[1, 512, 125, 63]	280,064
│ │ └─Bottleneck: 3	[1, 512, 125, 63]	280,064
│ └─Sequential: 3	[1, 1024, 125, 63]	--
│ │ └─Bottleneck: 0	[1, 1024, 125, 63]	1,512,448
│ │ └─Bottleneck: 1	[1, 1024, 125, 63]	1,117,184
│ │ └─Bottleneck: 2	[1, 1024, 125, 63]	1,117,184
│ │ └─Bottleneck: 3	[1, 1024, 125, 63]	1,117,184
│ │ └─Bottleneck: 4	[1, 1024, 125, 63]	1,117,184
│ │ └─Bottleneck: 5	[1, 1024, 125, 63]	1,117,184
│ │ └─Bottleneck: 6	[1, 1024, 125, 63]	1,117,184

│ │ └─Bottleneck: 7	[1, 1024, 125, 63]	1,117,184
│ │ └─Bottleneck: 8	[1, 1024, 125, 63]	1,117,184
│ │ └─Bottleneck: 9	[1, 1024, 125, 63]	1,117,184
│ │ └─Bottleneck: 10	[1, 1024, 125, 63]	1,117,184
│ │ └─Bottleneck: 11	[1, 1024, 125, 63]	1,117,184
│ │ └─Bottleneck: 12	[1, 1024, 125, 63]	1,117,184
│ │ └─Bottleneck: 13	[1, 1024, 125, 63]	1,117,184
│ │ └─Bottleneck: 14	[1, 1024, 125, 63]	1,117,184
│ │ └─Bottleneck: 15	[1, 1024, 125, 63]	1,117,184
│ │ └─Bottleneck: 16	[1, 1024, 125, 63]	1,117,184
│ │ └─Bottleneck: 17	[1, 1024, 125, 63]	1,117,184
│ │ └─Bottleneck: 18	[1, 1024, 125, 63]	1,117,184
│ │ └─Bottleneck: 19	[1, 1024, 125, 63]	1,117,184
│ │ └─Bottleneck: 20	[1, 1024, 125, 63]	1,117,184
│ │ └─Bottleneck: 21	[1, 1024, 125, 63]	1,117,184
│ │ └─Bottleneck: 22	[1, 1024, 125, 63]	1,117,184
│ └─Sequential: 4	[1, 2048, 125, 63]	--
│ │ └─Bottleneck: 0	[1, 2048, 125, 63]	6,039,552
│ │ └─Bottleneck: 1	[1, 2048, 125, 63]	4,462,592
│ │ └─Bottleneck: 2	[1, 2048, 125, 63]	4,462,592
├─FCNHead:	[1, 6, 125, 63]	--
│ └─Conv2d: 0	[1, 512, 125, 63]	9,437,184
│ └─BatchNorm2d: 1	[1, 512, 125, 63]	1,024
│ └─ReLU: 2	[1, 512, 125, 63]	--
│ └─Dropout: 3	[1, 512, 125, 63]	--
│ └─Conv2d: 4	[1, 6, 125, 63]	3,078

==
=========================

Total params: 51,941,446

Trainable params: 51,941,446

Non-trainable params: 0

Total mult-adds (G): 415.05

==
==========================

Input size (MB): 6.00

Forward/backward pass size (MB): 7395.96

Params size (MB): 207.77

Estimated Total Size (MB): 7609.73

==
==========================

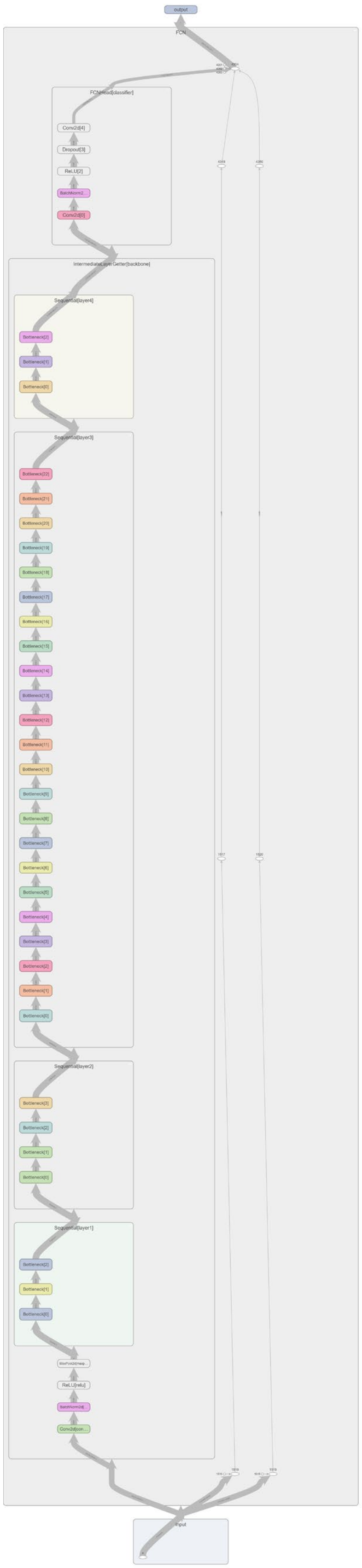

SI Figure 1: Generalized structure of a Pytorch implementation of a Fully Convolutional Network with a Res-net 101 backbone. (SVG image, please zoom in for details).

SI Figure 2: Detailed summary of the individual processes occurring in each layer of a Pytorch implementation of a Fully Convolutional Network with a Res-net 101 backbone.

```
FCN(
 (backbone): IntermediateLayerGetter(
  (conv1): Conv2d(3, 64, kernel_size=(7, 7), stride=(2, 2), padding=(3, 3), bias=False)
  (bn1): BatchNorm2d(64, eps=1e-05, momentum=0.1, affine=True, track_running_stats=True)
  (relu): ReLU(inplace=True)
  (maxpool): MaxPool2d(kernel_size=3, stride=2, padding=1, dilation=1, ceil_mode=False)
  (layer1): Sequential(
   (0): Bottleneck(
    (conv1): Conv2d(64, 64, kernel_size=(1, 1), stride=(1, 1), bias=False)
    (bn1): BatchNorm2d(64, eps=1e-05, momentum=0.1, affine=True,
track_running_stats=True)
    (conv2): Conv2d(64, 64, kernel_size=(3, 3), stride=(1, 1), padding=(1, 1), bias=False)
    (bn2): BatchNorm2d(64, eps=1e-05, momentum=0.1, affine=True,
track_running_stats=True)
    (conv3): Conv2d(64, 256, kernel_size=(1, 1), stride=(1, 1), bias=False)
    (bn3): BatchNorm2d(256, eps=1e-05, momentum=0.1, affine=True,
track_running_stats=True)
    (relu): ReLU(inplace=True)
    (downsample): Sequential(
     (0): Conv2d(64, 256, kernel_size=(1, 1), stride=(1, 1), bias=False)
     (1): BatchNorm2d(256, eps=1e-05, momentum=0.1, affine=True,
track_running_stats=True)
    )
   )
   (1): Bottleneck(
    (conv1): Conv2d(256, 64, kernel_size=(1, 1), stride=(1, 1), bias=False)
```

```
        (bn1): BatchNorm2d(64, eps=1e-05, momentum=0.1, affine=True,
track_running_stats=True)
        (conv2): Conv2d(64, 64, kernel_size=(3, 3), stride=(1, 1), padding=(1, 1), bias=False)
        (bn2): BatchNorm2d(64, eps=1e-05, momentum=0.1, affine=True,
track_running_stats=True)
        (conv3): Conv2d(64, 256, kernel_size=(1, 1), stride=(1, 1), bias=False)
        (bn3): BatchNorm2d(256, eps=1e-05, momentum=0.1, affine=True,
track_running_stats=True)
        (relu): ReLU(inplace=True)
      )
      (2): Bottleneck(
        (conv1): Conv2d(256, 64, kernel_size=(1, 1), stride=(1, 1), bias=False)
        (bn1): BatchNorm2d(64, eps=1e-05, momentum=0.1, affine=True,
track_running_stats=True)
        (conv2): Conv2d(64, 64, kernel_size=(3, 3), stride=(1, 1), padding=(1, 1), bias=False)
        (bn2): BatchNorm2d(64, eps=1e-05, momentum=0.1, affine=True,
track_running_stats=True)
        (conv3): Conv2d(64, 256, kernel_size=(1, 1), stride=(1, 1), bias=False)
        (bn3): BatchNorm2d(256, eps=1e-05, momentum=0.1, affine=True,
track_running_stats=True)
        (relu): ReLU(inplace=True)
      )
    )
    (layer2): Sequential(
      (0): Bottleneck(
        (conv1): Conv2d(256, 128, kernel_size=(1, 1), stride=(1, 1), bias=False)
        (bn1): BatchNorm2d(128, eps=1e-05, momentum=0.1, affine=True,
track_running_stats=True)
        (conv2): Conv2d(128, 128, kernel_size=(3, 3), stride=(2, 2), padding=(1, 1), bias=False)
        (bn2): BatchNorm2d(128, eps=1e-05, momentum=0.1, affine=True,
track_running_stats=True)
```

```
        (conv3): Conv2d(128, 512, kernel_size=(1, 1), stride=(1, 1), bias=False)
        (bn3): BatchNorm2d(512, eps=1e-05, momentum=0.1, affine=True,
track_running_stats=True)
        (relu): ReLU(inplace=True)
        (downsample): Sequential(
          (0): Conv2d(256, 512, kernel_size=(1, 1), stride=(2, 2), bias=False)
          (1): BatchNorm2d(512, eps=1e-05, momentum=0.1, affine=True,
track_running_stats=True)
        )
      )
      (1): Bottleneck(
        (conv1): Conv2d(512, 128, kernel_size=(1, 1), stride=(1, 1), bias=False)
        (bn1): BatchNorm2d(128, eps=1e-05, momentum=0.1, affine=True,
track_running_stats=True)
        (conv2): Conv2d(128, 128, kernel_size=(3, 3), stride=(1, 1), padding=(1, 1), bias=False)
        (bn2): BatchNorm2d(128, eps=1e-05, momentum=0.1, affine=True,
track_running_stats=True)
        (conv3): Conv2d(128, 512, kernel_size=(1, 1), stride=(1, 1), bias=False)
        (bn3): BatchNorm2d(512, eps=1e-05, momentum=0.1, affine=True,
track_running_stats=True)
        (relu): ReLU(inplace=True)
      )
      (2): Bottleneck(
        (conv1): Conv2d(512, 128, kernel_size=(1, 1), stride=(1, 1), bias=False)
        (bn1): BatchNorm2d(128, eps=1e-05, momentum=0.1, affine=True,
track_running_stats=True)
        (conv2): Conv2d(128, 128, kernel_size=(3, 3), stride=(1, 1), padding=(1, 1), bias=False)
        (bn2): BatchNorm2d(128, eps=1e-05, momentum=0.1, affine=True,
track_running_stats=True)
        (conv3): Conv2d(128, 512, kernel_size=(1, 1), stride=(1, 1), bias=False)
```

```
        (bn3): BatchNorm2d(512, eps=1e-05, momentum=0.1, affine=True,
track_running_stats=True)
        (relu): ReLU(inplace=True)
      )
      (3): Bottleneck(
        (conv1): Conv2d(512, 128, kernel_size=(1, 1), stride=(1, 1), bias=False)
        (bn1): BatchNorm2d(128, eps=1e-05, momentum=0.1, affine=True,
track_running_stats=True)
        (conv2): Conv2d(128, 128, kernel_size=(3, 3), stride=(1, 1), padding=(1, 1), bias=False)
        (bn2): BatchNorm2d(128, eps=1e-05, momentum=0.1, affine=True,
track_running_stats=True)
        (conv3): Conv2d(128, 512, kernel_size=(1, 1), stride=(1, 1), bias=False)
        (bn3): BatchNorm2d(512, eps=1e-05, momentum=0.1, affine=True,
track_running_stats=True)
        (relu): ReLU(inplace=True)
      )
    )
    (layer3): Sequential(
      (0): Bottleneck(
        (conv1): Conv2d(512, 256, kernel_size=(1, 1), stride=(1, 1), bias=False)
        (bn1): BatchNorm2d(256, eps=1e-05, momentum=0.1, affine=True,
track_running_stats=True)
        (conv2): Conv2d(256, 256, kernel_size=(3, 3), stride=(1, 1), padding=(1, 1), bias=False)
        (bn2): BatchNorm2d(256, eps=1e-05, momentum=0.1, affine=True,
track_running_stats=True)
        (conv3): Conv2d(256, 1024, kernel_size=(1, 1), stride=(1, 1), bias=False)
        (bn3): BatchNorm2d(1024, eps=1e-05, momentum=0.1, affine=True,
track_running_stats=True)
        (relu): ReLU(inplace=True)
        (downsample): Sequential(
          (0): Conv2d(512, 1024, kernel_size=(1, 1), stride=(1, 1), bias=False)
```

```
          (1): BatchNorm2d(1024, eps=1e-05, momentum=0.1, affine=True,
track_running_stats=True)
        )
      )
      (1): Bottleneck(
        (conv1): Conv2d(1024, 256, kernel_size=(1, 1), stride=(1, 1), bias=False)
        (bn1): BatchNorm2d(256, eps=1e-05, momentum=0.1, affine=True,
track_running_stats=True)
        (conv2): Conv2d(256, 256, kernel_size=(3, 3), stride=(1, 1), padding=(2, 2), dilation=(2, 2),
bias=False)
        (bn2): BatchNorm2d(256, eps=1e-05, momentum=0.1, affine=True,
track_running_stats=True)
        (conv3): Conv2d(256, 1024, kernel_size=(1, 1), stride=(1, 1), bias=False)
        (bn3): BatchNorm2d(1024, eps=1e-05, momentum=0.1, affine=True,
track_running_stats=True)
        (relu): ReLU(inplace=True)
      )
      (2): Bottleneck(
        (conv1): Conv2d(1024, 256, kernel_size=(1, 1), stride=(1, 1), bias=False)
        (bn1): BatchNorm2d(256, eps=1e-05, momentum=0.1, affine=True,
track_running_stats=True)
        (conv2): Conv2d(256, 256, kernel_size=(3, 3), stride=(1, 1), padding=(2, 2), dilation=(2, 2),
bias=False)
        (bn2): BatchNorm2d(256, eps=1e-05, momentum=0.1, affine=True,
track_running_stats=True)
        (conv3): Conv2d(256, 1024, kernel_size=(1, 1), stride=(1, 1), bias=False)
        (bn3): BatchNorm2d(1024, eps=1e-05, momentum=0.1, affine=True,
track_running_stats=True)
        (relu): ReLU(inplace=True)
      )
      (3): Bottleneck(
```

(conv1): Conv2d(1024, 256, kernel_size=(1, 1), stride=(1, 1), bias=False)

(bn1): BatchNorm2d(256, eps=1e-05, momentum=0.1, affine=True, track_running_stats=True)

(conv2): Conv2d(256, 256, kernel_size=(3, 3), stride=(1, 1), padding=(2, 2), dilation=(2, 2), bias=False)

(bn2): BatchNorm2d(256, eps=1e-05, momentum=0.1, affine=True, track_running_stats=True)

(conv3): Conv2d(256, 1024, kernel_size=(1, 1), stride=(1, 1), bias=False)

(bn3): BatchNorm2d(1024, eps=1e-05, momentum=0.1, affine=True, track_running_stats=True)

(relu): ReLU(inplace=True)

)

(4): Bottleneck(

(conv1): Conv2d(1024, 256, kernel_size=(1, 1), stride=(1, 1), bias=False)

(bn1): BatchNorm2d(256, eps=1e-05, momentum=0.1, affine=True, track_running_stats=True)

(conv2): Conv2d(256, 256, kernel_size=(3, 3), stride=(1, 1), padding=(2, 2), dilation=(2, 2), bias=False)

(bn2): BatchNorm2d(256, eps=1e-05, momentum=0.1, affine=True, track_running_stats=True)

(conv3): Conv2d(256, 1024, kernel_size=(1, 1), stride=(1, 1), bias=False)

(bn3): BatchNorm2d(1024, eps=1e-05, momentum=0.1, affine=True, track_running_stats=True)

(relu): ReLU(inplace=True)

)

(5): Bottleneck(

(conv1): Conv2d(1024, 256, kernel_size=(1, 1), stride=(1, 1), bias=False)

(bn1): BatchNorm2d(256, eps=1e-05, momentum=0.1, affine=True, track_running_stats=True)

(conv2): Conv2d(256, 256, kernel_size=(3, 3), stride=(1, 1), padding=(2, 2), dilation=(2, 2), bias=False)

```
        (bn2): BatchNorm2d(256, eps=1e-05, momentum=0.1, affine=True,
track_running_stats=True)
        (conv3): Conv2d(256, 1024, kernel_size=(1, 1), stride=(1, 1), bias=False)
        (bn3): BatchNorm2d(1024, eps=1e-05, momentum=0.1, affine=True,
track_running_stats=True)
        (relu): ReLU(inplace=True)
      )
      (6): Bottleneck(
        (conv1): Conv2d(1024, 256, kernel_size=(1, 1), stride=(1, 1), bias=False)
        (bn1): BatchNorm2d(256, eps=1e-05, momentum=0.1, affine=True,
track_running_stats=True)
        (conv2): Conv2d(256, 256, kernel_size=(3, 3), stride=(1, 1), padding=(2, 2), dilation=(2, 2),
bias=False)
        (bn2): BatchNorm2d(256, eps=1e-05, momentum=0.1, affine=True,
track_running_stats=True)
        (conv3): Conv2d(256, 1024, kernel_size=(1, 1), stride=(1, 1), bias=False)
        (bn3): BatchNorm2d(1024, eps=1e-05, momentum=0.1, affine=True,
track_running_stats=True)
        (relu): ReLU(inplace=True)
      )
      (7): Bottleneck(
        (conv1): Conv2d(1024, 256, kernel_size=(1, 1), stride=(1, 1), bias=False)
        (bn1): BatchNorm2d(256, eps=1e-05, momentum=0.1, affine=True,
track_running_stats=True)
        (conv2): Conv2d(256, 256, kernel_size=(3, 3), stride=(1, 1), padding=(2, 2), dilation=(2, 2),
bias=False)
        (bn2): BatchNorm2d(256, eps=1e-05, momentum=0.1, affine=True,
track_running_stats=True)
        (conv3): Conv2d(256, 1024, kernel_size=(1, 1), stride=(1, 1), bias=False)
        (bn3): BatchNorm2d(1024, eps=1e-05, momentum=0.1, affine=True,
track_running_stats=True)
        (relu): ReLU(inplace=True)
```

```
)
(8): Bottleneck(
  (conv1): Conv2d(1024, 256, kernel_size=(1, 1), stride=(1, 1), bias=False)
  (bn1): BatchNorm2d(256, eps=1e-05, momentum=0.1, affine=True,
track_running_stats=True)
  (conv2): Conv2d(256, 256, kernel_size=(3, 3), stride=(1, 1), padding=(2, 2), dilation=(2, 2),
bias=False)
  (bn2): BatchNorm2d(256, eps=1e-05, momentum=0.1, affine=True,
track_running_stats=True)
  (conv3): Conv2d(256, 1024, kernel_size=(1, 1), stride=(1, 1), bias=False)
  (bn3): BatchNorm2d(1024, eps=1e-05, momentum=0.1, affine=True,
track_running_stats=True)
  (relu): ReLU(inplace=True)
)
(9): Bottleneck(
  (conv1): Conv2d(1024, 256, kernel_size=(1, 1), stride=(1, 1), bias=False)
  (bn1): BatchNorm2d(256, eps=1e-05, momentum=0.1, affine=True,
track_running_stats=True)
  (conv2): Conv2d(256, 256, kernel_size=(3, 3), stride=(1, 1), padding=(2, 2), dilation=(2, 2),
bias=False)
  (bn2): BatchNorm2d(256, eps=1e-05, momentum=0.1, affine=True,
track_running_stats=True)
  (conv3): Conv2d(256, 1024, kernel_size=(1, 1), stride=(1, 1), bias=False)
  (bn3): BatchNorm2d(1024, eps=1e-05, momentum=0.1, affine=True,
track_running_stats=True)
  (relu): ReLU(inplace=True)
)
(10): Bottleneck(
  (conv1): Conv2d(1024, 256, kernel_size=(1, 1), stride=(1, 1), bias=False)
  (bn1): BatchNorm2d(256, eps=1e-05, momentum=0.1, affine=True,
track_running_stats=True)
```

(conv2): Conv2d(256, 256, kernel_size=(3, 3), stride=(1, 1), padding=(2, 2), dilation=(2, 2), bias=False)

(bn2): BatchNorm2d(256, eps=1e-05, momentum=0.1, affine=True, track_running_stats=True)

(conv3): Conv2d(256, 1024, kernel_size=(1, 1), stride=(1, 1), bias=False)

(bn3): BatchNorm2d(1024, eps=1e-05, momentum=0.1, affine=True, track_running_stats=True)

(relu): ReLU(inplace=True)

)

(11): Bottleneck(

(conv1): Conv2d(1024, 256, kernel_size=(1, 1), stride=(1, 1), bias=False)

(bn1): BatchNorm2d(256, eps=1e-05, momentum=0.1, affine=True, track_running_stats=True)

(conv2): Conv2d(256, 256, kernel_size=(3, 3), stride=(1, 1), padding=(2, 2), dilation=(2, 2), bias=False)

(bn2): BatchNorm2d(256, eps=1e-05, momentum=0.1, affine=True, track_running_stats=True)

(conv3): Conv2d(256, 1024, kernel_size=(1, 1), stride=(1, 1), bias=False)

(bn3): BatchNorm2d(1024, eps=1e-05, momentum=0.1, affine=True, track_running_stats=True)

(relu): ReLU(inplace=True)

)

(12): Bottleneck(

(conv1): Conv2d(1024, 256, kernel_size=(1, 1), stride=(1, 1), bias=False)

(bn1): BatchNorm2d(256, eps=1e-05, momentum=0.1, affine=True, track_running_stats=True)

(conv2): Conv2d(256, 256, kernel_size=(3, 3), stride=(1, 1), padding=(2, 2), dilation=(2, 2), bias=False)

(bn2): BatchNorm2d(256, eps=1e-05, momentum=0.1, affine=True, track_running_stats=True)

(conv3): Conv2d(256, 1024, kernel_size=(1, 1), stride=(1, 1), bias=False)

(bn3): BatchNorm2d(1024, eps=1e-05, momentum=0.1, affine=True, track_running_stats=True)

(relu): ReLU(inplace=True)

)

(13): Bottleneck(

(conv1): Conv2d(1024, 256, kernel_size=(1, 1), stride=(1, 1), bias=False)

(bn1): BatchNorm2d(256, eps=1e-05, momentum=0.1, affine=True, track_running_stats=True)

(conv2): Conv2d(256, 256, kernel_size=(3, 3), stride=(1, 1), padding=(2, 2), dilation=(2, 2), bias=False)

(bn2): BatchNorm2d(256, eps=1e-05, momentum=0.1, affine=True, track_running_stats=True)

(conv3): Conv2d(256, 1024, kernel_size=(1, 1), stride=(1, 1), bias=False)

(bn3): BatchNorm2d(1024, eps=1e-05, momentum=0.1, affine=True, track_running_stats=True)

(relu): ReLU(inplace=True)

)

(14): Bottleneck(

(conv1): Conv2d(1024, 256, kernel_size=(1, 1), stride=(1, 1), bias=False)

(bn1): BatchNorm2d(256, eps=1e-05, momentum=0.1, affine=True, track_running_stats=True)

(conv2): Conv2d(256, 256, kernel_size=(3, 3), stride=(1, 1), padding=(2, 2), dilation=(2, 2), bias=False)

(bn2): BatchNorm2d(256, eps=1e-05, momentum=0.1, affine=True, track_running_stats=True)

(conv3): Conv2d(256, 1024, kernel_size=(1, 1), stride=(1, 1), bias=False)

(bn3): BatchNorm2d(1024, eps=1e-05, momentum=0.1, affine=True, track_running_stats=True)

(relu): ReLU(inplace=True)

)

(15): Bottleneck(

(conv1): Conv2d(1024, 256, kernel_size=(1, 1), stride=(1, 1), bias=False)

(bn1): BatchNorm2d(256, eps=1e-05, momentum=0.1, affine=True, track_running_stats=True)

(conv2): Conv2d(256, 256, kernel_size=(3, 3), stride=(1, 1), padding=(2, 2), dilation=(2, 2), bias=False)

(bn2): BatchNorm2d(256, eps=1e-05, momentum=0.1, affine=True, track_running_stats=True)

(conv3): Conv2d(256, 1024, kernel_size=(1, 1), stride=(1, 1), bias=False)

(bn3): BatchNorm2d(1024, eps=1e-05, momentum=0.1, affine=True, track_running_stats=True)

(relu): ReLU(inplace=True)

)

(16): Bottleneck(

(conv1): Conv2d(1024, 256, kernel_size=(1, 1), stride=(1, 1), bias=False)

(bn1): BatchNorm2d(256, eps=1e-05, momentum=0.1, affine=True, track_running_stats=True)

(conv2): Conv2d(256, 256, kernel_size=(3, 3), stride=(1, 1), padding=(2, 2), dilation=(2, 2), bias=False)

(bn2): BatchNorm2d(256, eps=1e-05, momentum=0.1, affine=True, track_running_stats=True)

(conv3): Conv2d(256, 1024, kernel_size=(1, 1), stride=(1, 1), bias=False)

(bn3): BatchNorm2d(1024, eps=1e-05, momentum=0.1, affine=True, track_running_stats=True)

(relu): ReLU(inplace=True)

)

(17): Bottleneck(

(conv1): Conv2d(1024, 256, kernel_size=(1, 1), stride=(1, 1), bias=False)

(bn1): BatchNorm2d(256, eps=1e-05, momentum=0.1, affine=True, track_running_stats=True)

(conv2): Conv2d(256, 256, kernel_size=(3, 3), stride=(1, 1), padding=(2, 2), dilation=(2, 2), bias=False)

(bn2): BatchNorm2d(256, eps=1e-05, momentum=0.1, affine=True, track_running_stats=True)

(conv3): Conv2d(256, 1024, kernel_size=(1, 1), stride=(1, 1), bias=False)

(bn3): BatchNorm2d(1024, eps=1e-05, momentum=0.1, affine=True, track_running_stats=True)

(relu): ReLU(inplace=True)

)

(18): Bottleneck(

(conv1): Conv2d(1024, 256, kernel_size=(1, 1), stride=(1, 1), bias=False)

(bn1): BatchNorm2d(256, eps=1e-05, momentum=0.1, affine=True, track_running_stats=True)

(conv2): Conv2d(256, 256, kernel_size=(3, 3), stride=(1, 1), padding=(2, 2), dilation=(2, 2), bias=False)

(bn2): BatchNorm2d(256, eps=1e-05, momentum=0.1, affine=True, track_running_stats=True)

(conv3): Conv2d(256, 1024, kernel_size=(1, 1), stride=(1, 1), bias=False)

(bn3): BatchNorm2d(1024, eps=1e-05, momentum=0.1, affine=True, track_running_stats=True)

(relu): ReLU(inplace=True)

)

(19): Bottleneck(

(conv1): Conv2d(1024, 256, kernel_size=(1, 1), stride=(1, 1), bias=False)

(bn1): BatchNorm2d(256, eps=1e-05, momentum=0.1, affine=True, track_running_stats=True)

(conv2): Conv2d(256, 256, kernel_size=(3, 3), stride=(1, 1), padding=(2, 2), dilation=(2, 2), bias=False)

(bn2): BatchNorm2d(256, eps=1e-05, momentum=0.1, affine=True, track_running_stats=True)

(conv3): Conv2d(256, 1024, kernel_size=(1, 1), stride=(1, 1), bias=False)

(bn3): BatchNorm2d(1024, eps=1e-05, momentum=0.1, affine=True, track_running_stats=True)

(relu): ReLU(inplace=True)

```
)
(20): Bottleneck(
(conv1): Conv2d(1024, 256, kernel_size=(1, 1), stride=(1, 1), bias=False)
(bn1): BatchNorm2d(256, eps=1e-05, momentum=0.1, affine=True,
track_running_stats=True)
(conv2): Conv2d(256, 256, kernel_size=(3, 3), stride=(1, 1), padding=(2, 2), dilation=(2, 2),
bias=False)
(bn2): BatchNorm2d(256, eps=1e-05, momentum=0.1, affine=True,
track_running_stats=True)
(conv3): Conv2d(256, 1024, kernel_size=(1, 1), stride=(1, 1), bias=False)
(bn3): BatchNorm2d(1024, eps=1e-05, momentum=0.1, affine=True,
track_running_stats=True)
(relu): ReLU(inplace=True)
)
(21): Bottleneck(
(conv1): Conv2d(1024, 256, kernel_size=(1, 1), stride=(1, 1), bias=False)
(bn1): BatchNorm2d(256, eps=1e-05, momentum=0.1, affine=True,
track_running_stats=True)
(conv2): Conv2d(256, 256, kernel_size=(3, 3), stride=(1, 1), padding=(2, 2), dilation=(2, 2),
bias=False)
(bn2): BatchNorm2d(256, eps=1e-05, momentum=0.1, affine=True,
track_running_stats=True)
(conv3): Conv2d(256, 1024, kernel_size=(1, 1), stride=(1, 1), bias=False)
(bn3): BatchNorm2d(1024, eps=1e-05, momentum=0.1, affine=True,
track_running_stats=True)
(relu): ReLU(inplace=True)
)
(22): Bottleneck(
(conv1): Conv2d(1024, 256, kernel_size=(1, 1), stride=(1, 1), bias=False)
(bn1): BatchNorm2d(256, eps=1e-05, momentum=0.1, affine=True,
track_running_stats=True)
```

```
        (conv2): Conv2d(256, 256, kernel_size=(3, 3), stride=(1, 1), padding=(2, 2), dilation=(2, 2),
bias=False)
        (bn2): BatchNorm2d(256, eps=1e-05, momentum=0.1, affine=True,
track_running_stats=True)
        (conv3): Conv2d(256, 1024, kernel_size=(1, 1), stride=(1, 1), bias=False)
        (bn3): BatchNorm2d(1024, eps=1e-05, momentum=0.1, affine=True,
track_running_stats=True)
        (relu): ReLU(inplace=True)
      )
    )
    (layer4): Sequential(
      (0): Bottleneck(
        (conv1): Conv2d(1024, 512, kernel_size=(1, 1), stride=(1, 1), bias=False)
        (bn1): BatchNorm2d(512, eps=1e-05, momentum=0.1, affine=True,
track_running_stats=True)
        (conv2): Conv2d(512, 512, kernel_size=(3, 3), stride=(1, 1), padding=(2, 2), dilation=(2, 2),
bias=False)
        (bn2): BatchNorm2d(512, eps=1e-05, momentum=0.1, affine=True,
track_running_stats=True)
        (conv3): Conv2d(512, 2048, kernel_size=(1, 1), stride=(1, 1), bias=False)
        (bn3): BatchNorm2d(2048, eps=1e-05, momentum=0.1, affine=True,
track_running_stats=True)
        (relu): ReLU(inplace=True)
        (downsample): Sequential(
          (0): Conv2d(1024, 2048, kernel_size=(1, 1), stride=(1, 1), bias=False)
          (1): BatchNorm2d(2048, eps=1e-05, momentum=0.1, affine=True,
track_running_stats=True)
        )
      )
      (1): Bottleneck(
        (conv1): Conv2d(2048, 512, kernel_size=(1, 1), stride=(1, 1), bias=False)
```

```
      (bn1): BatchNorm2d(512, eps=1e-05, momentum=0.1, affine=True, track_running_stats=True)
      (conv2): Conv2d(512, 512, kernel_size=(3, 3), stride=(1, 1), padding=(4, 4), dilation=(4, 4), bias=False)
      (bn2): BatchNorm2d(512, eps=1e-05, momentum=0.1, affine=True, track_running_stats=True)
      (conv3): Conv2d(512, 2048, kernel_size=(1, 1), stride=(1, 1), bias=False)
      (bn3): BatchNorm2d(2048, eps=1e-05, momentum=0.1, affine=True, track_running_stats=True)
      (relu): ReLU(inplace=True)
    )
    (2): Bottleneck(
      (conv1): Conv2d(2048, 512, kernel_size=(1, 1), stride=(1, 1), bias=False)
      (bn1): BatchNorm2d(512, eps=1e-05, momentum=0.1, affine=True, track_running_stats=True)
      (conv2): Conv2d(512, 512, kernel_size=(3, 3), stride=(1, 1), padding=(4, 4), dilation=(4, 4), bias=False)
      (bn2): BatchNorm2d(512, eps=1e-05, momentum=0.1, affine=True, track_running_stats=True)
      (conv3): Conv2d(512, 2048, kernel_size=(1, 1), stride=(1, 1), bias=False)
      (bn3): BatchNorm2d(2048, eps=1e-05, momentum=0.1, affine=True, track_running_stats=True)
      (relu): ReLU(inplace=True)
    )
  )
 )
 (classifier): FCNHead(
  (0): Conv2d(2048, 512, kernel_size=(3, 3), stride=(1, 1), padding=(1, 1), bias=False)
  (1): BatchNorm2d(512, eps=1e-05, momentum=0.1, affine=True, track_running_stats=True)
  (2): ReLU()
  (3): Dropout(p=0.1, inplace=False)
```

```
    (4): Conv2d(512, 6, kernel_size=(1, 1), stride=(1, 1))
  )
)
```
